# *De novo* Transcriptome Analysis of Drought-Adapted Cluster Bean (Cultivar RGC-1025) Reveals the Wax Regulatory Genes Involved in Drought Resistance

**DOI:** 10.3389/fpls.2022.868142

**Published:** 2022-06-28

**Authors:** B. Manohara Reddy, A. M. Anthony Johnson, N. Jagadeesh Kumar, Boya Venkatesh, N. Jayamma, Merum Pandurangaiah, Chinta Sudhakar

**Affiliations:** ^1^Plant Molecular Biology Laboratory, Department of Botany, Sri Krishnadevaraya University, Anantapur, India; ^2^Department of Biotechnology, St. Josephs College (Autonomous), Bengaluru, India

**Keywords:** drought stress, transcriptome, wax genes, cluster bean (*Cyamopsis tetragonoloba* L.), differentailly expressed genes

## Abstract

Cluster bean (*Cyamopsis tetragonoloba* L.) is one of the multipurpose underexplored crops grown as green vegetable and for gum production in dryland areas. Cluster bean is known as relatively tolerant to drought and salinity stress. To elucidate the molecular mechanisms involved in the drought tolerance of cluster bean cultivar RGC-1025, RNA sequencing (RNA-seq) of the drought-stressed and control samples was performed. *De novo* assembly of the reads resulted in 66,838 transcripts involving 203 pathways. Among these transcripts, differentially expressed gene (DEG) analysis resulted in some of the drought-responsive genes expressing *alpha dioxygenase* 2, low temperature-induced 65 kDa protein (LDI65), putative vacuolar amino acid transporter, and late embryogenesis abundant protein (LEA 3). The analysis also reported drought-responsive transcription factors (TFs), such as NAC, WRKY, GRAS, and MYB families. The relative expression of genes by qRT-PCR revealed consistency with the DEG analysis. Key genes involved in the wax biosynthesis pathway were mapped using the DEG data analysis. These results were positively correlated with epicuticular wax content and the wax depositions on the leaf surfaces, as evidenced by scanning electron microscope (SEM) image analysis. Further, these findings support the fact that enhanced wax deposits on the leaf surface had played a crucial role in combating the drought stress in cluster beans under drought stress conditions. In addition, this study provided a set of unknown genes and TFs that could be a source of engineering tolerance against drought stress in cluster beans.

## Introduction

*Cyamopsis tetragonoloba* (L.) Taub. (Cluster bean) is a drought-adapted annual legume crop with lower water requirements than many other dryland legume crops. Cluster beans can grow in marginal soils because of their high water use efficiency, deep tap rooting system, etc. In India, cluster bean is cultivated for its green vegetables, foraging cattle, green manure, and dry pods for guar gum production (Rao and Shahid, 2011; Global Agricultural Information Network [GAIN], 2014). Globally, India ranks first and produces about 80% of the world’s cluster beans, and Rajasthan is the top state, making 75% of the total production in India. Due to high prices and export demand for guar gum, the cultivation of cluster bean is gradually increased in India from the year 2010 onward, and the total area is about 5,345 ha with 615 kg per ha yield during the agricultural year 2018–2019 (DoA, Government of India, Annual Report). The guar gum produced from cluster beans is rich in galactomannan, which is 78–82% of the seed’s endosperm. Guar gum is an essential non-toxic agrochemical, mostly an export product, and a source of polysaccharide emulsifier used primarily in the food, cosmetic, and pharmaceutical sectors (Mudgil et al., 2014). In addition, it is also used in the oil and gas industry as a gelling agent and as an additive in the milling industry (Coveney et al., 2000). The co-products for guar are guar meal and guar bagasse used to produce biofuels and other value co-products (Gresta et al., 2017).

Although cluster bean is considered a highly valued crop, its productivity is lesser than other legume crops; consequently, a significant gap exists between demand and export of guar. Biotic and abiotic factors are major limiting factors for guar yield enhancement. Thus, there is a need to increase the productivity of cluster beans to meet the demand-supply gap of cluster beans and their derivatives through genetic enhancement (Kumar et al., 2020). To increase guar output, it is necessary to produce cluster bean cultivars with improved abiotic stress tolerance, particularly drought resistance, for growing in the semi-arid tropics. The genetic improvement of cluster beans for enhanced drought resistance is not achieved due to insufficient genomic resources and inadequate germplasm availability. *C. tetragonoloba* genome size has been estimated to be approximately 580 Mbp using flow cytometry (Tyagi et al., 2019). The present study adopted next-generation sequencing (NGS) technologies to understand the detailed information of the drought-stressed transcriptome of cluster beans. This technology enables the identification of differentially expressed genes (DEGs), the deciphering of metabolic pathways involved in drought resistance, gum biosynthesis, and the identification of DNA-based markers, all of which may open up new avenues for molecular breeding to improve cluster bean production, gum quality, and stress resistance.

For the past few decades, extensive research has been carried out on applying omics technologies to identify many candidate genes, proteins, and metabolic pathways of various crop species under different stress conditions (Panda et al., 2021; Raza et al., 2021). In recent years, transcriptome technology has become an essential tool for analyzing the molecular mechanisms of abiotic stresses in plants (Cai et al., 2019; Hasan et al., 2019). Global transcriptome profiling of drought-stressed grain legumes, such as chickpea (Garg et al., 2011; Hiremath et al., 2011; Kumar et al., 2019), groundnut (Brasileiro et al., 2015; Zhao et al., 2018), and lentil (Singh et al., 2017; Morgil et al., 2019), revealed a set of DEGs involved in various metabolic pathways under stress conditions. Following transcriptome analysis, Wu et al. (2016) have identified 22 NAC TFs from drought-tolerant and drought-sensitive genotypes of common bean. Transcriptome analysis of drought-tolerant and drought-sensitive genotypes of wheat showed significant induction or repression of genes involved in secondary metabolism, nucleic acid synthesis, protein synthesis, and transport in the tolerant genotype when compared with the sensitive genotype (Kumar et al., 2018). RNA sequencing (RNA-Seq) analysis has been employed to elucidate drought-tolerance molecular mechanisms in other crops, such as cotton (Hasan et al., 2019), buckwheat (Hou et al., 2019), and Proso millet (Zhang et al., 2019). To date, very few studies on the transcriptome analysis of *C. tetragonoloba* have been published. For instance, Rawal et al. (2017) reported an RNA-Seq-based transcriptome from the leaf, shoot, and flower tissues of Guar; Tanwar et al. (2017) detailed the transcriptome of leaf tissues from two leaf tissue guar varieties M-83 and RGC-1066. Al-Qurainy et al. (2019) published a transcriptome of guar, accession BWP 5595 under various treatments, such as drought, salinity, and heat stress. The present study was focused on targeted transcriptome deep sequencing of a drought-adapted cultivar RGC-1025 to characterize the genes responsible for the drought resistance. The gene information thus obtained would pave the way for using DEGs in developing strategies for drought resistance through various approaches. Moreover, transcriptome data sets could be valuable for novel gene discovery and the marker-assisted selective breeding of cluster bean species.

## Materials and Methods

### Screening Cluster Bean Cultivars for Drought Tolerance

Initially, four cluster bean cultivars, namely, RGC-1025, RGC-1038, RGC-1055, and RGC-1066, were screened for their drought tolerance based on various parameters, such as germination, seedling growth, biomass, relative water content (Barrs and Weatherley, 1962), cell membrane injury (Leopold et al., 1981), malondialdehyde (MDA) (Hodges et al., 1999), total chlorophylls (Arnon, 1949), and total proline content (Bates et al., 1973).

### Plant Samples, Processing, and Sequencing

Seeds of cluster bean (*C. tetragonoloba* L.) cultivar RGC-1025 were sterilized in 0.5% (W/V) sodium hypochlorite solution for 5 min, then rinsed thoroughly, and soaked in distilled water for 30 min. Seeds were sown in earthen pots containing soil and farmyard manure in a 3:1 proportion maintained in the departmental botanical garden. After 20 days post-sowing, drought stress was induced by withholding water to one set of pots, and respective fully watered controls were maintained in another set of pots. Ten days after stress imposition, fresh leaf samples from five plants were collected, pooled, flash-frozen in liquid nitrogen, and transported immediately to the sequencing facility.

For total RNA-seq, total RNA was extracted using the Qiagen RNeasy Plant Mini Kit with DNAse treatment (Thermo Fisher Scientific, United States) as per the manufacturer’s instructions. The quality and quantity of the RNA were estimated using a NanoDrop Spectrophotometer (Thermo Fisher Scientific, United States) and Qubit Fluorometer (Thermo Fisher Scientific, United States). The integrity of the RNA samples was analyzed using Agilent 2100 Bioanalyzer (Agilent Technologies, Santa Clara, CA, United States). RNA-seq libraries were prepared with Illumina-compatible NEB Next^®^ Ultra™ II Directional RNA Library Prep Kit (New England BioLabs, MA, United States). In total, 500 ng of total RNA was taken for mRNA isolation, fragmentation, and priming. Fragmented and primed mRNA was subjected to first-strand synthesis followed by second-strand synthesis. The double-stranded cDNA was purified using JetSeq Clean Beads (Bioline Meridian Bioscience, Australia). Purified cDNA was end-repaired, adenylated, and Illumina multiplex barcode adapters were ligated as per NEBNext^®^ Ultra™ II Directional RNA Library Prep protocol, followed by second-strand excision using USER enzyme at 37°C for 15 min. Adapter-ligated cDNA was purified using JetSeq Beads and was subjected to 10 cycles for indexing (98°C for 30 s, cycling (98°C for 10 s, 65°C for 75 s) and 65°C for 5 min) to enrich the adapter-ligated fragments. The final PCR product (sequencing library) was purified with JetSeq Beads, followed by a library-quality control check using Agilent 2100 Bioanalyzer (Agilent Technologies, Santa Clara, CA, United States). A total of 7,629,816 short reads were obtained, 150 bp length paired-end (P.E.) reads and average fragment size of ∼400 bp were used, and three biological replicates were processed for sequencing analysis with Illumina HiSeq™ 4000, which was out-sourced at Genotypic Technologies, Bengaluru, India.

### Read Quality Control, Adapter Removal, *de novo* Assembly, and Clustering

The reads were processed for quality assessment and low-quality filtering before the FastQC tool assembly. The reads were then processed by removing the adapter sequences and low-quality bases (<q30) using the Cutadapt tool. Processed reads were assembled using a graph-based approach by the rnaSPAdes program. The characteristic properties, such as N50 length, average length, maximum length, and a minimum length of the assembled contigs, were calculated. *De novo* transcriptome assembly of the processed reads from all the libraries was done using Bowtie2 with end-to-end parameters. In the second step of the assembly procedure, clustering of the assembled transcripts based on sequence similarity is performed using the Cluster Database at High Identity with Tolerance (CD-HIT)-EST program^1^ with 95% similarity between the sequences. This reduces the redundancy without excluding sequence diversity used for further transcript annotation and the DEG analysis.

### Functional Annotation of Transcripts

All unigenes were annotated using the BLASTX search tool on *Viridiplantae* transcripts from the UniProt database containing 8,058,045 protein sequences and the NCBI non-redundant database (N.R.). The cutoff e-value was 10^––5,^ and the minimum similarity was more significant than 40%. Gene ontology annotation was carried out using the Blast2go program and visualized using Web Gene Ontology Annotation Plot (WEGO).^2^

### Differentially Expressed Gene Analysis and Pathway Analysis

DESeq, an R package, was used for differential expression analysis. Sequencing (variable library size/depth) bias among the samples was removed by library normalization using size factor calculation in DESeq. DESeq normalized expression values were used to calculate fold change for a given transcript. The regulation for each transcript was assigned based on log2-fold change. The transcripts that show a log2-fold change less than −1 are represented as downregulated. The values greater than one are upregulated and between −1 and 1 are termed neutrally regulated. Gene Ontology (GO) enrichment analysis and pathway analysis for DEG were done against the Kyoto Encyclopedia of Genes and Genomes (KEGG) database. KAAS server was used to analyze and characterize associated pathways. To obtain the highly significant differential expression genes, the criterion of the absolute value of reads per kilobase of transcript per million reads (RPKM) ratio > 1,000 was used.

### Mining of Simple Sequence Repeats

Simple sequence repeats (SSRs) were identified using the MISA Perl script in each transcript (MIcroSAtellite identification tool).^3^ A simple repetition of motif length ranging from 1 to 6 bp was identified with recommended default parameters of MISA.

### qRT-PCR Analysis of Gene Expression

To evaluate the gene expression pattern from the DEG analysis, the total RNA extracted, as mentioned earlier, from control and drought-stressed cluster bean variety RGC-1025 was used for the SYBR Green qRT-PCR assay. The qRT-PCR assay was performed for 16 different stress-responsive genes selected from DEG analysis. These genes include *aldo-keto reductase 1* (*AKR1*), *late embryogenesis Abundant14* (*LEA14*), *non-specific lipid transfer protein*, TFs *MYB30*, *NAC4*, *scare crow-like protein1* (GRAS TF’s), *BHLH*, *GATA*, *malate dehydrogenase* (*MDH*), *aquaporin*, *DNA helicase*, *nitrate reductase*, *proline dehydrogenase* (*PRODH*), *serine hydroxy methyltransferase* (*SHMT*), and *thaumatin, trehalose 6-phosphate phosphatase* (*TRE6PH*) with actin and tubulin genes as an internal control. For cDNA synthesis, 1 μg of total RNA from control and drought-stressed cluster bean RGC-1025 samples was treated with a Turbo DNASE treatment kit (Thermo Fisher Scientific, United States) as per the manufacturer’s protocol to remove any DNA traces. cDNA was synthesized using Revert Aid M-MuLV Reverse Transcriptase (Thermo Fisher Scientific) as per the manufacturer’s instructions. qRT-PCR mix was comprised of 1× using Power SYBR Green Master Mix (Ambion, United States), 20 ng of cDNA, and 0.2 μM of forward and reverse primers. Supplementary Table 1 shows the primers used for the investigated genes. The RT-PCR analysis was done on Applied Biosystems Step One Real-Time PCR machine with standard cycling comprising 95°C for 30 s, 40 cycles of 95°C for 1 s, 60°C for 20 s, and a melt curve analysis. Relative quantification was studied using 2^–Δ^ ^Δ^ CT method (Livak and Schmittgen, 2001). Each gene was analyzed in three biological samples, and three reaction replicates were performed for each biological sample.

### Estimation of Epicuticular Wax and Scanning Electron Microscope Imaging of Leaf Surfaces for Wax Deposits

Leaf surface waxes exteriorly deposited were extracted and quantified by a colorimetric assay reported by Mamrutha et al. (2010). Carnauba wax was used as the standard for the wax quantification assay. The wax content is represented as μg/gm fresh weight.

An scanning electron microscope (SEM) examined the epicuticular wax crystals. The third and fourth leaves of drought-stressed and control plants were cut to 0.5 cm, mounted onto standard stubs, and coated with gold particles using a fully automated vacuum spotter smart coater (DII-29030SCTR, JOEL, United States). The surfaces of the coated samples were observed through an SEM (JOEL-JSM-IT500, Japan).

## Results

### Screening Cluster Bean Cultivars for Drought Tolerance

Cluster bean cultivars, namely, RGC-1025, RGC-1038, RGC-1055, and RGC-1066, were screened for drought tolerance and we found significant differences at the cultivar level in morphological and biochemical traits under stress treatments. Results revealed that among four cultivars evaluated; cultivar RGC-1025 showed a lesser decrease in seedling growth, better biomass, and relative water content; lesser extent of cell membrane injury, lesser MDA, total chlorophylls content, and significantly higher levels of osmoprotectant, proline when compared to other cultivars (Supplementary Tables), suggesting the relative tolerance of cultivar RGC-1025 over other cultivars to drought stress. Therefore, we further extended transcriptome studies to understand the molecular mechanisms conferring the drought tolerance of cultivar RGC-1025.

### Reads and *de novo* Assembly

To construct the transcriptome of cluster bean cultivar RGC-1025, high-quality RNAs from three replicates of drought-stressed and unstressed conditions (control) were sequenced. An average of 18 million paired-end reads were used for the downstream analysis after pre-processing. In total, 76,129,816 short reads were obtained using Illumina HiSeq 4000 Technology (Table 1). Most of the reads had > 99.9% score. Around 97.5% of the reads were retained in both the samples post-filtering. The cleaned-up reads were assembled using the Bowtie2 tool. CD-HIT was used to cluster redundant and similar isoforms. Finally, 66,838 transcripts were clustered with an average length of 955 bp. The non-redundant transcripts were considered as unigenes and were further analyzed.

**TABLE 1:** Sample wise assembly statistics of cluster bean cultivar RGC-1025 samples.

S. no	Samples	Control	Stressed
1	Raw reads	43,110,222	33,019,594
2	Processed reads	42,051,780	32,208,984
3	Percentage of reads retained	97.5%	97.5%
4	Alignment to clustered transcripts (%)	89.00%	90.65%
5	Number of transcripts identified	123,594	106,025
6	Maximum contig length	21,802	15,999
7	Minimum contig length	31	31
8	Average contig length	489.6	521.9
9	Median contig length	261	266
10	Total contigs length	6,050,9220	55,334,262
11	Total number of non-ATGC characters	5,596	4,419
12	Contigs ≥ 100 bp	123,411	105,933
13	Contigs ≥ 200 bp	97,982	84762
14	Contigs ≥ 500 bp	26,684	25,767
15	Contigs ≥ 1 Kbp	15,221	14,891
16	Contigs ≥ 10 Kbp	5	7
17	Contigs ≥ 1 Mbp	0	0
18	N50 value	875	1003

### Characterization of Unigenes

**FIGURE 1:**
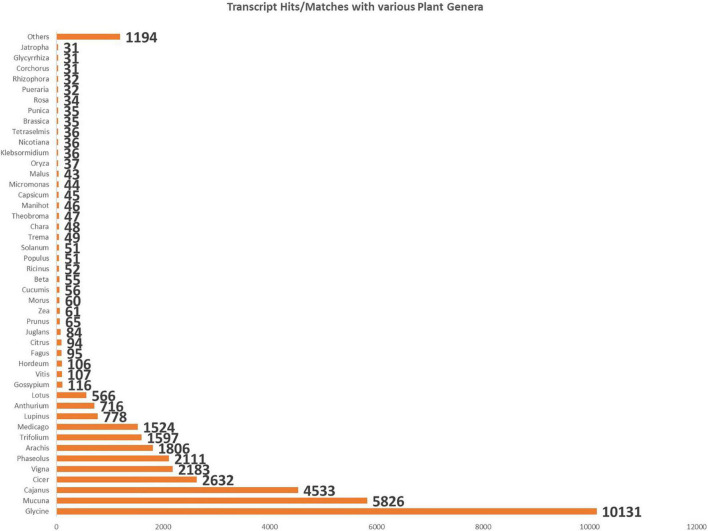
Bar chart shows the number of BlastX hits of cluster bean cultivar RGC-1025 with maximum hits from members of the family Fabaceae.

All the obtained unigenes were annotated against the Uniprot Viridiplantae sequence database, NCBI non-redundant database, with a cutoff E-value of 10^–5^. Around 55.98% of the unigenes were found to have hits in the public databases. The transcripts with more than 30% identity were considered during the analysis. These unigenes were classified as 31 functional categories. The significant fall under the category of DNA templated transcriptional regulation (2.34%) among the biological processes, the category of integral component of the membrane (25.4%) under cellular components, and the category of adenosine 5′-triphosphate (ATP) binding (13.14%) under molecular function. The highest transcript matches during the functional annotation with members of the family Fabaceae, such as *Glycine* (10,131), *Mucuna* (5,826), *Cajanus* (4,533), *Cicer* (2,632), *Vigna* (2,183), *Phaseolus* (2,111), *Arachis* (1,806), *Trifolium* (1,597), and *Medicago* (1,524) (Figure 1).

### Functional Classification

**FIGURE 2:**
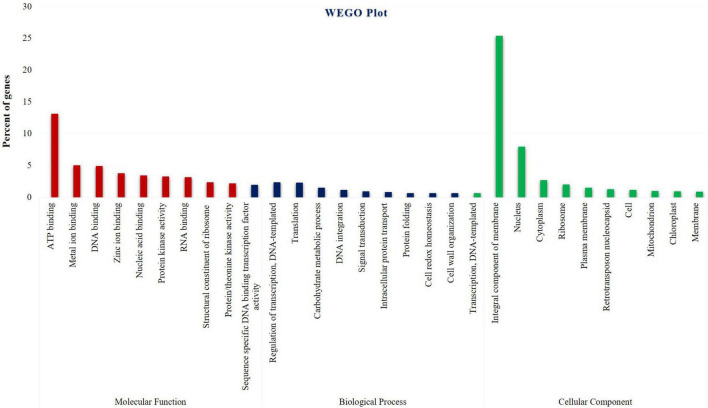
Pie chart shows drought-stressed cluster bean cultivar RGC-1025 Gene Ontology classification of transcripts into biological process, molecular functions, and cellular components.

Differentially expressed genes were subjected to GO analysis to achieve functional classification. As a result, 37,418 DEGs fall into (i) molecular function, (ii) biological process, and iii) cellular components. In total, 30,900 (50.2%) DEGs were associated with molecular function terms, such as ATP binding encoding transcripts, followed by metal ion binding transcripts, DNA binding transcripts, zinc ion binding transcripts, nucleic acid binding transcripts, protein kinase activity transcripts, etc., and 16,410 (26.6%) DEGs were annotated with cellular component terms, represented by the integral component of the membrane encoding transcripts, followed by nucleus components transcripts, cytoplasm components transcripts, ribosome transcripts, plasma membrane transcripts, retrotransposon nucleocapsid transcripts, etc., and 14,191 (23%) DEGs were associated with biological process terms. This study also observed that identical DEG sequences could exist in more than one category. The most represented “biological process” subcategories identified were DNA-templated transcriptional regulation encoding transcripts followed by translation components, carbohydrate metabolic process transcripts, DNA integration transcripts, signal transduction transcripts, intracellular protein transcripts, etc. (Figure 2 and Supplementary Tables 2–4). In general, 12,866 genes were found to be upregulated, 16,177 genes were downregulated, and 27,782 genes had shown no change in their expression levels in cluster bean cultivar RGC-1025 due to drought stress. More interestingly, 3,745 transcripts were expressed only in the stressed sample.

### Differentially Expressed Genes in Kyoto Encyclopedia of Genes and Genomes Pathways

**FIGURE 3:**
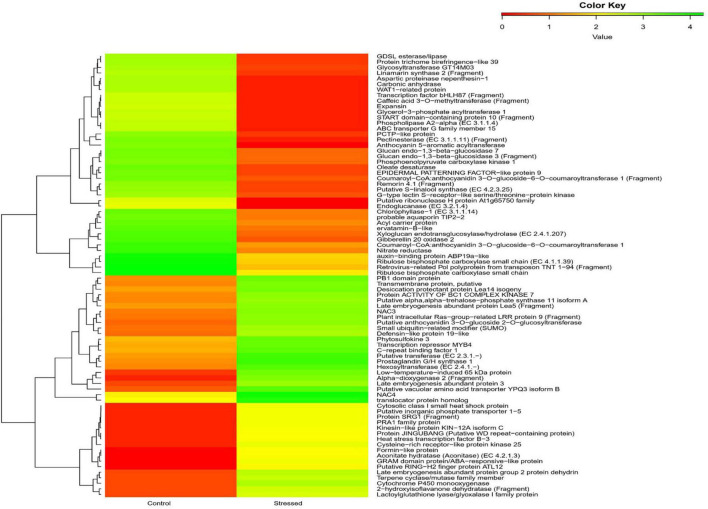
Heatmap shows the top 40 upregulated and downregulated genes of cluster bean cultivar RGC-1025 under control and drought stress.

Kyoto Encyclopedia of Genes and Genomes is an online database that deals with genomes and enzymatic pathways, and its identifiers were looked to predict biochemical pathways related to DEGs. Among 66,838 transcripts in unigene pathways, 17,211 transcripts against the KEGG pathways were identified. Of the 203 pathways identified, the top forty pathways are shown in Supplementary Table 5. DESeq analysis of the transcripts revealed that the enzymes with the most frequency of expression in the category of upregulated genes were alpha dioxygenase 2 (9.4-fold), low temperature-induced 65 kDa protein (LTI65; 9.2-fold), putative vacuolar amino acid transporter (9.05-fold), hexosyl transferase (EC 2.4.1.-; 7.95 fold), late embryogenesis abundant protein3 (LEA 3; 7.79-fold), Putative anthocyanidin 3-O-glucoside 2″-O-glucosyltransferase (EC 2.4.1.297; 7.44-fold), Glucosyltransferases, Rab-like GTPase Activators and Myotubularins (GRAM) domain protein/abscisic acid (ABA)-responsive-like protein (putative GRAM domain, P.H. domain-containing protein) (7.30-fold), and cytochrome P450 monooxygenase (EC:1.14.14.80; 7.14-fold). In the category of downregulated genes, the genes encoding the following proteins were found to be downregulated, such as putative CDP-alcohol phosphatidyl transferase class-I family protein 3 (EC 2.7.8.1; 0.5-fold), NEDD4-binding protein 2 (0.49-fold), dihydrolipoamide acetyltransferase component of pyruvate dehydrogenase complex (EC 2.3.1.-; 0.49-fold), putative cyclic nucleotide-gated ion channel 14 (0.498), ATP binding cassette (ABC) transporter G family member 28 (0.49-fold), N-(5-phosphoribosyl) anthranilate isomerase (putative phosphoribosyl anthranilate isomerase) (EC 5.3.1.24; 0.49-fold), F-box/FBD/LRR-repeat protein (0.49-fold), and adenylate kinase (EC:2.7.4.3; 0.49-fold). The heat map of DEGs showed the genes expressed (Figure 3).

### Differentially Expressed Transcription Factors

**FIGURE 4:**
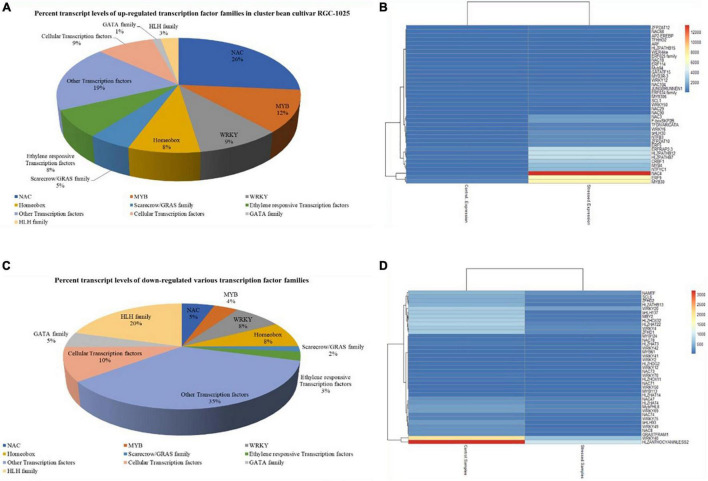
**(A)** Upregulated transcription factor families of drought-stressed cluster bean cultivar RGC-1025. **(B)** Heatmap of the top 40 upregulated transcription factors of cluster bean cultivar RGC-1025 in drought stress. **(C)** Downregulated transcription factor families of drought-stressed cluster bean cultivar RGC-1025. **(D)** Heatmap of the top 40 downregulated transcription factors of cluster bean RGC-1025 in drought stress.

Among the drought stress-responsive upregulated TF families, NAC family TF was the most abundant (26%), followed by MYB TFs (12%) and WRKY TFs (9%) (Figure 4A). Figure 4B depicts the top 40 upregulated TFs under drought treatments in cluster bean cultivar RGC-1025. Most renowned drought stress-responsive TFs upregulated include NAC4, NAC3, NAC29, NAC 104, and NAC18 from the NAC family, followed by WRKY12, WRKY50, WRKY6, WRKY33, WRKY30, WRKY24, WRKY42, WRKY53, WRKY70, and WRKY7 from WRKY family, further followed by other TFs, such as homeobox domain TFs, scarecrow/GRAS TFs, and ethylene-responsive TFs. Among the downregulated TF families, the hemophagocytic lymphohistiocytosis (HLH) TF family was the most abundant (20%), followed by the cellular TF family (10%) and homeobox domains (∼8%) (Figures 4C,D).

### Simple Sequence Repeat Mining

**FIGURE 5:**
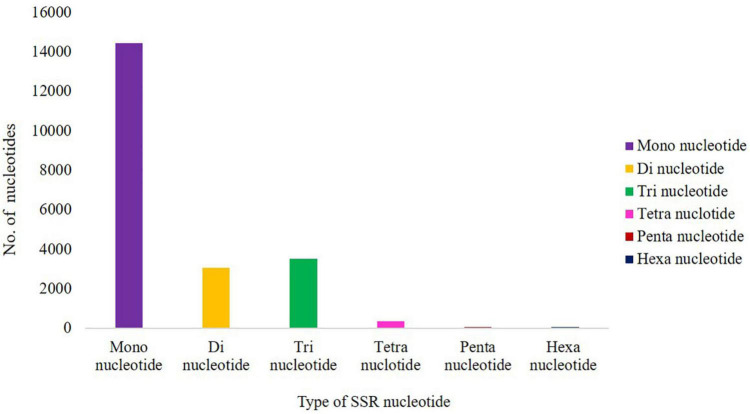
Distribution of different classes of SSRs in cluster bean cultivar RGC-1025.

A total of 21,494 SSRs were identified in the cluster bean data, 14,434 (67.15%) are mono-nucleotide repeats, 3,072 (14.29%) are di-nucleotide repeats, 3,516 (16.35%) are tri-nucleotide repeats, 341 (1.58%) tetra-nucleotide repeats, 69 (0.32%) pentanucleotide repeats, and 63 (0.25%) hexanucleotide repeats (Figure 5). The 21,494 potential SSRs identified from *de novo* transcriptome sequencing data represent a significant addition to the limited set of genic-SSR markers available in cluster bean cultivar RGC-1025.

### Validation of Differentially Expressed Genes by Quantitative Real-Time RT-PCR

**FIGURE 6:**
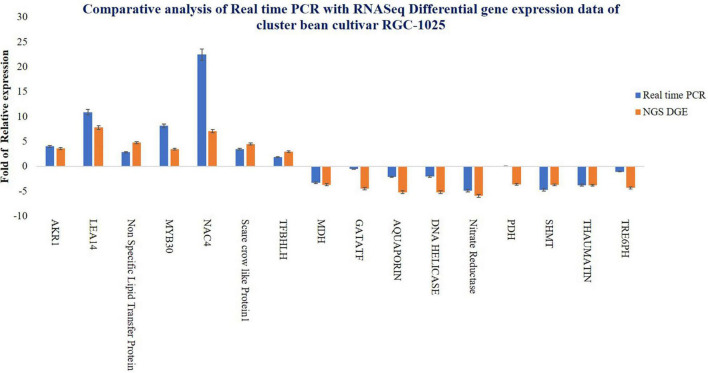
Comparison of real-time PCR data and RNA-Seq differential gene expression data of cluster bean cultivar RGC-1025.

To verify the reliability of the expression profiles from RNAseq data, qRT-PCR analysis was performed for DEG (Figure 6). Results of the qRT-PCR assay for 16 stress-responsive genes revealed consistency in gene expression patterns as compared to that of the DEG analysis of the transcriptome of RGC-1025. The relative fold change of both the qRT-PCR and NGS-DEG is represented (Figure 6). Upregulation of stress-responsive TFs, such as NAC4, MYB30, scarecrow-like protein (SCL-1), primary helix-loop-helix TF (SlbHLH22), and TF bHLH 22, and the overexpression of candidate stress-responsive functional genes, such as DNA helicase, MDH, AKR1, LEA14, PDH, and SHMT, during drought stress by improving the ROS scavenging system, increasing osmotic potential, stomatal regulation, pH stability, respiration, and β-oxidation of fatty acids could support further the drought tolerance of cluster bean cultivar RGC-1025 (Ahmad et al., 2017; Waseem et al., 2019). The correlation among the expression patterns of the genes in NGS and qRT-PCR represents the consistency of the data in the current study.

### Epicuticular Wax Content and Scanning Electron Microscope Imaging of Leaf Surfaces

**FIGURE 7:**
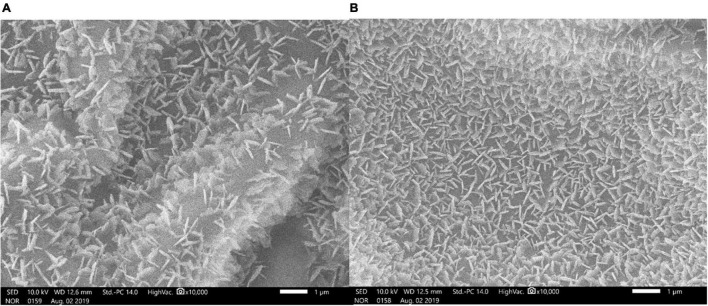
Scanning electron microscopy (SEM) analysis (2,000 × with 20 μm bar scale) of epicuticular wax depositions on the leaf surfaces of cluster bean cultivar RGC-1025 **(A)** control leaf and **(B)** drought-stressed leaf.

The epicuticular wax content of the cluster bean cultivar RGC-1025 during the drought stress was 610.36 ± 0.53 μg/dm^2^ as compared to the control 432.41 ± 0.4 μg/dm^2^, which is 41.15% higher as compared to its control. These epicuticular wax data were supported by the SEM imaging of the leaf surfaces of control and drought-stressed plants. The SEM image of the leaf surface of RGC-1025 under drought stress showed enhanced wax crystal deposits (Figure 7).

### Mapping of the Wax Biosynthesis Pathway in Cluster Bean Using Differentially Expressed Gene Data

**FIGURE 8:**
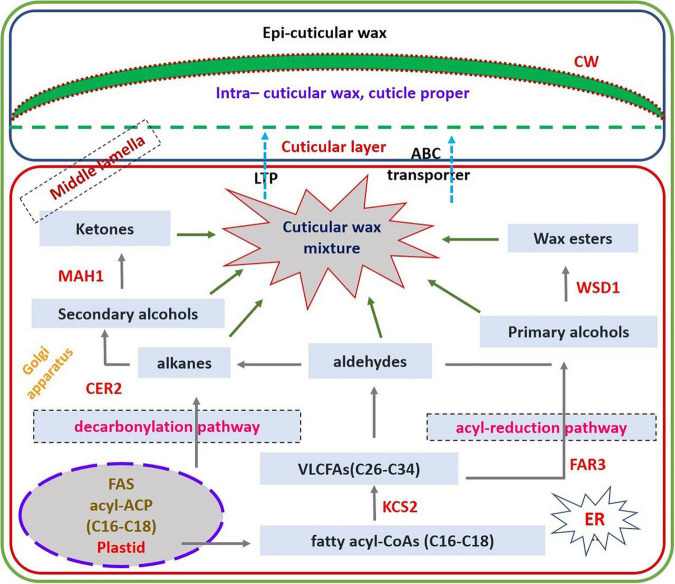
Upregulated genes involved in Wax biosynthesis pathway.

Among the genes differentially expressed in wax biosynthesis pathway, there were upregulated genes that include *KCS1* that encodes β-ketoacyl-CoA synthase 1 that is involved in elongation of 24C fatty acids, *WSD1* that encodes wax ester synthase/diacylglycerol acyl transferase, which involves in wax ester biosynthesis, *KCR1* that encodes β-Ketoacyl-CoA reductase, which is involved in very long-chain fatty acid elongation (VLFCA elongation), FATB that encodes acyl-acyl carrier protein thioesterase, which is engaged in supply of saturated fatty acids for wax biosynthesis, *CER4/FAR3* that encodes alcohol forming fatty acyl CoA reductase, which is involved in formation of C24:0 and C26:0 primary alcohols, protein WAX2 encoding gene, CER17 also called Eceriferum1, which encodes for acyl-CoA desaturase-like 4 protein that is involved in n-6 desaturation of very long-chain acyl-CoAs, ABC transporter G family member 11, which encodes ABC transporter proteins that is involved in secretion of surface waxes in interaction with CER5, which is an another ABC transporter protein, and lipid transfer protein gene that encodes a lipid transport protein, which has role in cuticular wax export or accumulation. Finally, the upregulation of these wax genes in the present study through transcriptome DEG data reveals that these gene products are responsible for accumulating or producing epicuticular wax in cluster bean cultivar RGC-1025 (Figure 8). qRT-PCR analysis of selective wax genes showed significant changes in the expression patterns and an increase in the expression of the KCS1 gene was 1.85-fold, WSD1 gene was 1.81-fold, KCR1gene was 3.73-fold, FATB gene was 1.8-fold, CER4/FAR3 gene was 0.4-fold, protein WAX2 gene was 2.36-fold, CER17 gene was 2.89-fold, ABC transporter G family member11 gene was 1.95-fold, and lipid transfer protein gene was 1.49-fold.

## Discussion

Cluster bean (*C. tetragonoloba* L.) is an annual legume crop grown in arid and semiarid regions. Due to the lack of genomic resources, presently, conventional breeding is the only means of cluster bean improvement. In this regard, the availability of genomic resources can serve as a good platform for cluster bean improvement (Naoumkina et al., 2007; Tanwar et al., 2017). Cluster bean is known as relatively tolerant to abiotic stresses. Genotypic variation in stress tolerance exists in cluster bean cultivars (Alshameri et al., 2017), implying that it is a valuable repository for genes that are resistant to these abiotic stresses, and to use this genetic tank, the present study implemented a *de novo* transcriptome analysis of a drought-tolerant cluster bean cultivar RGC-1025.

The RNA-Seq (NGS) method offers a holistic view of the transcriptome, revealing many novel transcribed regions, splice isoforms, genic microsatellites, and the precise location of transcription boundaries (Cloonan et al., 2008; Wang et al., 2009; Li et al., 2010; Wilhelm et al., 2010). These technologies have been widely exploited in numerous plant species to produce molecular markers using transcriptome analysis (Dutta et al., 2011; Wang et al., 2014). In the present study, Illumina HiSeq 4000 Technology generated 76,129,816 short reads from the control and drought-stressed samples of cluster bean cultivar RGC-1025.

A cluster bean is a non-model plant without prior genome knowledge; BLASTX was used to search for sequence similarity and compare the assembled unigenes of the cluster bean transcriptome against multiple databases. Around 55.98% of the unigenes were obtained and annotated against the Uniprot Viridiplantae sequence database and NCBI non-redundant database, with a cutoff E-value of 10^–5^. According to species distribution analyses, many plant species have sequences that are homologous to cluster bean sequences. The highest transcript matches during the functional annotation with members of the family Fabaceae, such as *Glycine* (10131), *Mucuna* (5826), and *Cajanus* (4533).

Gene Ontology analysis provides a set of dynamically controlled and structured vocabularies for describing the roles of genes in any organism (Ashburner et al., 2000). Based on the sequence homology, 37,418 DEGs were assigned GO terms and classified into three categories, namely, molecular function, biological process, and cellular components. The results of this study agree with those of other plant leaf transcriptome investigations (Wu et al., 2015; Bose Mazumdar and Chattopadhyay, 2016). Alpha-dioxygenase (-DOX) is engaged in the catalysis of fatty acid oxygenation, resulting in the production of a recently found category of oxylipins, which plays a crucial role in shielding tissues from oxidative damage and cell death under drought stress (Tirajoh et al., 2005). Shi et al. (2015) reported that LTI30 protein positively regulates drought stress resistance in Arabidopsis through the modulation of ABA sensitivity, hydrogen peroxide levels, and proline accumulation. Yang et al. (2015) reported from their study that putative cationic amino acid transporter 9 (CAT9) mutation resulted in chlorotic leaves and overexpression resulted in the formation of stems and inflorescence transgenic *Arabidopsis* plants. Magwanga et al. (2018) also established the role of LEA proteins in cotton drought stress tolerance. Zheng et al. (2020) reported that the overexpressing phenotype of *Oryza sativa* ABA responsive protein 1 (OsABAR1), a GRAM protein-containing protein, showed resistance to drought and salinity. Identifying many DEGs in this study could help to gain in-depth knowledge of the diverse metabolic activities involved in the stress-resistant mechanisms of cluster beans. According to the gene function analysis, the KEGG database revealed that among 66,838 transcripts, 17,211 transcripts were allocated to 203 unigene pathways. A similar pattern was discovered in the transcriptome of *Phyllanthus amarus* leaves (Bose Mazumdar and Chattopadhyay, 2016).

Transcription factors are regulatory proteins involved in various regulatory processes, such as biotic and abiotic stress adaptation (Nakashima et al., 2014; Joshi et al., 2016). TF genes, such as NAC, WRKY, MYB, and bZIP, have been linked to drought stress responses (Gahlaut et al., 2016). NAC genes are TFs specific to plants and are involved in growth, development, and stress responses. Shi et al. (2018) reported that *GmWRKY12* confers drought and salt tolerance in soybean. Auxins usually induce scarecrow-like genes and interact with histone deacetylase, resulting in chromatin modeling in drought stress (Gao et al., 2004; Sánchez et al., 2007). Similarly, Scarecrow-like protein 1, one of the GRAS proteins, was upregulated in this study during drought stress. Zhu et al. (2014) studied the role of the *SlNAC4* TF in combating drought and salinity stress through RNAi-silenced transgenic tomato plants. Yu et al. (2016) also proved that *Cicer arietinum NAC4 (CarNAC4)* TF overexpression in *Arabidopsis* conferred resistance to drought and salinity stresses. Liu et al. (2013) reported enhanced dehydration and drought tolerance through overexpression of *AhNAC3* in tobacco through enhanced superoxide scavenging. Tang et al. (2017) reported that the overexpression of the peanut *NAC4* gene conferred drought tolerance in tobacco. These differentially expressed TFs propose their significant role in combating drought stress in cluster bean cultivar RGC-1025. SSRs are the most useful molecular markers for genetics and plant breeding applications (Hiremath et al., 2012). In the present study, 21,494 SSRs were identified in the cluster bean data, and the frequency distribution of SSR markers agrees with previous reports in guar (Kuravadi et al., 2014; Kumar et al., 2016).

Cuticular wax prevents non-stomatal water loss, allowing plants to adapt to water-limited conditions (Kerstiens, 1996; Buda et al., 2013). Cuticular waxes deposited on the plant’s organs play a critical role in sustaining harsh environmental conditions, such as drought (Jenks and Ashworth, 1999; Goodwin and Jenks, 2005). Drought stress enhances the increased deposition of waxes in many plants (Bondada et al., 1996; Samdur et al., 2003; Cameron et al., 2006). Lee and Suh (2013) and Mamrutha et al. (2017) reported that wax biosynthesis and its pathway genes are regulated at transcriptional, post-transcriptional, and translational levels. Guo et al. (2016) showed that drought-induced accumulation of wax biosynthesis positively correlated with drought-tolerant crops, such as wheat. In the present study, the ECERIFERUM1 was upregulated by 7.82-fold during the drought stress, revealing the upregulation of the wax biosynthesis pathway. Bourdenx et al. (2011) reported that overexpression of ECERIFERUM1 promotes wax’s very long-chain alkane biosynthesis and influences plant response to biotic and abiotic stresses. Xu et al. (2003) showed that an ABC transporter family gene, *AtTGD1*, is involved in the inter-organelle lipid transfer in Arabidopsis. Mizuno et al. (2013) reported that an ABC transporter gene, *Sb06g023280*, is responsible for epi-cuticular wax biosynthesis in Sorghum. Elango et al. (2020) assessed the epicuticular wax variability in the extensive genetic pool of Sorghum, and a genome-wide association mapping study showed genic regions associated with epicuticular wax production. Hence, the enhanced epicuticular wax content and the deposition of wax crystals on the leaf surfaces are essential components of plants for enhanced drought tolerance to overcome non-stomatal water loss.

## Conclusion

In summary, the Cluster bean cultivar RGC-1025 is proved to have enhanced drought tolerance that was evident from DEGs and analyzed from the transcriptome sequencing. The transcriptome sequencing and analysis revealed that the differential expression of the different stress responsible and constitutive cellular TFs, the enhanced traits, such as enhanced wax biosynthesis, and the upregulation of various genes involved in wax biosynthesis played a key role in RGC-1025 to combat drought stress efficiently.

## Data Availability Statement

The datasets presented in this study can be found in online repositories. The names of the repository/repositories and accession number(s) can be found below: https://www.ncbi.nlm.nih.gov/, #PRJNA669348.

## Author Contributions

CS conceptualized and supervised this study and wrote the manuscript. BR performed the experiments. BR, AA, and MP analyzed the transcriptome data. NJ, BV, and NJ performed RT PCR analysis. All authors equally contributed to manuscript revision, read, and approved the manuscript.

## Conflict of Interest

The authors declare that the research was conducted in the absence of any commercial or financial relationships that could be construed as a potential conflict of interest.

## Publisher’s Note

All claims expressed in this article are solely those of the authors and do not necessarily represent those of their affiliated organizations, or those of the publisher, the editors and the reviewers. Any product that may be evaluated in this article, or claim that may be made by its manufacturer, is not guaranteed or endorsed by the publisher.

## Footnotes

1
http:www.bioinformatics.org/cd-hit/

2
http://wego.genomics.org.cn/cgi-bin/wego/index.pl

3
http://pgrx.ipk-gatersleben.de/misa/
