# Supplementary material for: De novo Transcriptome Analysis of Drought-Adapted Cluster Bean (Cultivar RGC-1025) Reveals the Wax Regulatory Genes Involved in Drought Resistance
Source: Front Plant Sci. 2022 Jun 28;13:868142. doi: 10.3389/fpls.2022.868142 (PMC9274130; doi:10.3389/fpls.2022.868142)
Supplement: Supplementary file 1 [file Table_1.docx]

**Supplementary materials**

**
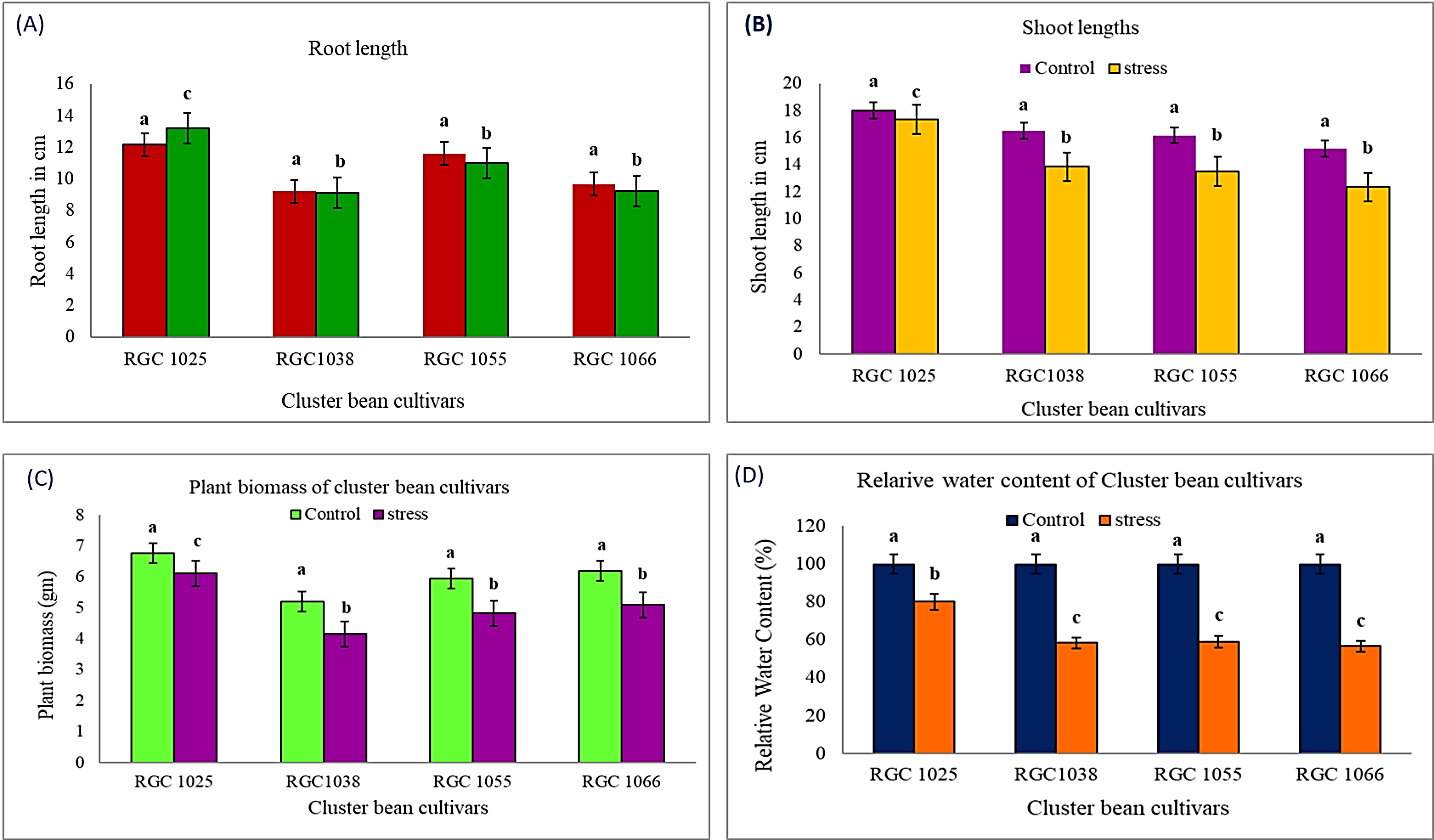
**

**Supplementary figure 1:** (A) Root length (cm) (B) shoot length (cm) (C) Plant dry weight (DW) (D) RWC (%) of four Cluster bean cultivars under control drought stress conditions.


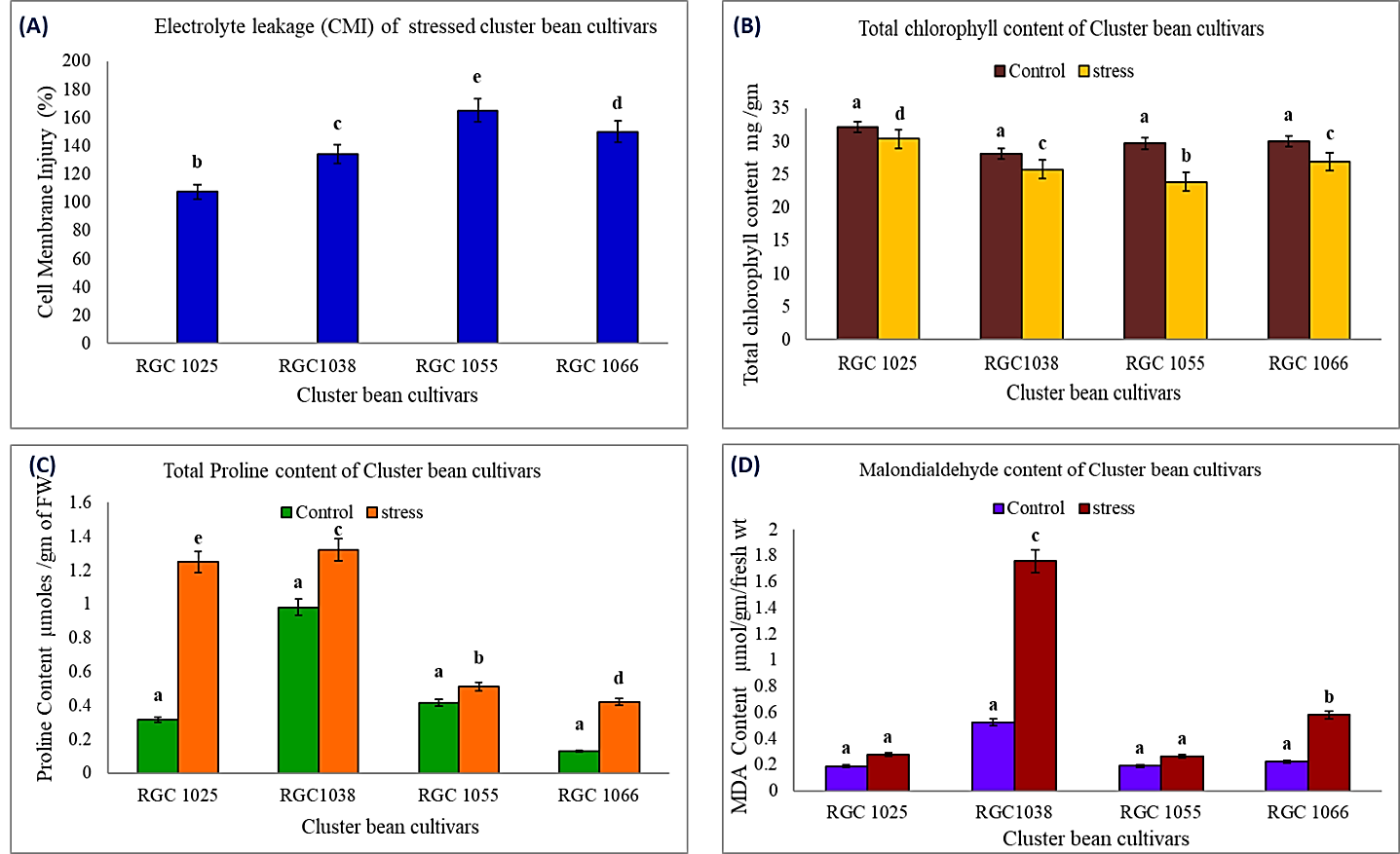


**Supplementary figure 2:** (A) Cell membrane injury (B) Total chlorophyll content (mg g^­^¹ FW) (C) Total Proline content (µmole g^­^¹ FW) (D) MDA content (n mole g^-1^ FW) of four Cluster bean cultivars under control drought stress conditions.


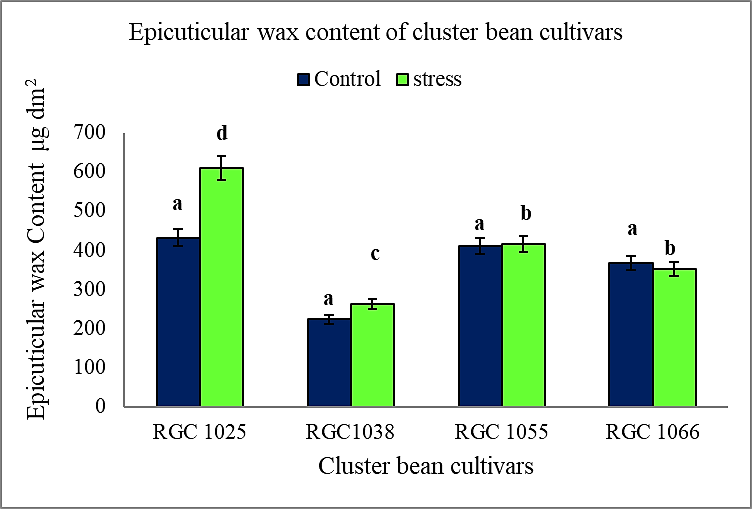


**Supplementary figure 3:** Epicuticular wax content (µg/dm^2^) of four cluster bean cultivars under control drought stress conditions.

**Supplementary Table 1: Primers used in qRT-PCR analysis**

| **S. No** | **Up-regulated genes** | **Sequence** | |
| --- | --- | --- | --- |
| 1 | AKR-1 | F | GGTCGCGGCTTTTTTGG |
|  |  | R | TGGAAGCGCGGATGATAATT |
| 2 | Lea14 | F | TGGGACATTGACTATCAACTAGATGTG |
|  |  | R | CCTTATGAGAAAGAGGAATGGTGAA |
| 3 | Non-Specific Lipid Transfer proteins | F | CAATGCTGCCGCCAAAAC |
|  |  | R | GCAGCGGATTTCATGCAGTT |
| 4 | MYB30 | F | AAACAGGTAATTGTGAAGGCAACA |
|  |  | R | CCCACTGCCCTCTTGGAA |
| 5 | NAC4 | F | GGAATCGCTTCCCGAAATC |
|  |  | R | GCGTTCTCAGCGAATTGACA |
| 6 | Scarecrow-like protein 1 | F | CCAGATTTGTTGAGGCCTACAAG |
|  |  | R | CCTGTCCTGACTCCCTCTAGGA |
| 7 | Transcription factor bHLH30 | F | AGAGGAGGAGAAGGGAGAGAATTAA |
|  |  | R | CAATTCCTTCACTTGGCTAACAACT |
| 8 | Malate Dehydrogenase (MDH) | F | CAACCCAATATCCAGATGTTAACCA |
|  |  | R | GCCAGTTATCATCAGCAATTGC |
| **Down-regulated Genes** | | | |
| 9 | GATA transcription factor | F | GCTTGTGGCGTTCGTTACAA |
|  |  | R | AGGACTAGCAGCAGGGCGATA |
| 10 | Aquaporin TIP2-1 | F | GGCTTGGCTGGCCTTATCTA |
|  |  | R | AACCTCAACCAAATCAAAATTCACT |
| 11 | DNA helicase | F | GGTTGAATGATGCTGACATGGA |
|  |  | R | TTGTCCGTGCGAAGATTCC |
| 12 | Nitrate reductase | F | TCTCAAACCACGCGAGAAAA |
|  |  | R | GCCTAACGTCGTGGGAAATAAC |
| 13 | Proline dehydrogenase | F | TGCAATATGTCCCATGAGTCTGT |
|  |  | R | CTCGCTTCCAGGGCAGATT |
| 14 | Serine hydroxymethyl transferase | F | GGCACACATCAGTGGATTGG |
|  |  | R | TGGTGGTCACAACATCTGCAT |
| 15 | Thaumatin-like protein 1 | F | AACCGGCTGTACTTCGGATCT |
|  |  | R | GCTTCCGTCTCCTGTTTTCAA |
| 16 | Trehalose 6-phosphate phosphatase | F | CTAGTGTTCCAAAAGAAACAGATGCT |
|  |  | R | AAACGCCGCAAAAATTCCT |
| **Internal Control Genes** | | | |
| 17 | Actin | F | CCGTCGCCACCGTTATCT |
|  |  | R | TGACTACGGCAGGACGAGAA |
| 18 | β-Tubulin | F | CGTGCCATATCCTCGAATCC |
|  |  | R | GCCTTCGCCGCAGAAA |

**Supplementary Table 2-** Top 40 transcripts Gene Ontology statistics of **Molecular function** in cluster bean cultivar RGC-1025.

| S. No | GO_ID | GO Description | Clustered Transcripts |
| --- | --- | --- | --- |
| 1 | GO:0005524 | ATP binding | 3407 |
| 2 | GO:0046872 | Metal ion binding | 1309 |
| 3 | GO:0003677 | DNA binding | 1280 |
| 4 | GO:0008270 | Zinc ion binding | 974 |
| 5 | GO:0003676 | Nucleic acid binding | 888 |
| 6 | GO:0004672 | Protein kinase activity | 842 |
| 7 | GO:0003723 | RNA binding | 825 |
| 8 | GO:0003735 | Structural constituent of ribosome | 620 |
| 9 | GO:0004674 | Protein serine/threonine kinase activity | 567 |
| 10 | GO:0003700 | Sequence-specific DNA binding transcription factor activity | 509 |
| 11 | GO:0016787 | Hydrolase activity | 444 |
| 12 | GO:0016491 | Oxidoreductase activity | 397 |
| 13 | GO:0043531 | ADP binding | 387 |
| 14 | GO:0005525 | GTP binding | 376 |
| 15 | GO:0022857 | Transmembrane transporter activity | 356 |
| 16 | GO:0020037 | Heme binding | 349 |
| 17 | GO:0005509 | Calcium ion binding | 321 |
| 18 | GO:0005506 | Iron ion binding | 315 |
| 19 | GO:0046983 | Protein dimerization activity | 305 |
| 20 | GO:0003824 | Catalytic activity | 279 |
| 21 | GO:0003924 | GTPase activity | 272 |
| 22 | GO:0043565 | Sequence-specific DNA binding | 267 |
| 23 | GO:0016874 | Ligase activity | 264 |
| 24 | GO:0008168 | Methyltransferase activity | 254 |
| 25 | GO:0016301 | Kinase activity | 245 |
| 26 | GO:0016705 | Oxidoreductase activity, acting on paired donors, with incorporation or reduction of molecular oxygen | 214 |
| 27 | GO:0016740 | Transferase activity | 203 |
| 28 | GO:0030246 | Carbohydrate binding | 200 |
| 29 | GO:0004497 | Monooxygenase activity | 194 |
| 30 | GO:0004553 | Hydrolase activity, hydrolyzing O-glycosyl compounds | 180 |
| 31 | GO:0008017 | Microtubule binding | 174 |
| 32 | GO:0000287 | Magnesium ion binding | 166 |
| 33 | GO:0016887 | ATPase activity | 164 |
| 34 | GO:0004386 | Helicase activity | 162 |
| 35 | GO:0009055 | Electron carrier activity | 160 |
| 36 | GO:0004842 | Ubiquitin-protein transferase activity | 158 |
| 37 | GO:0016757 | Transferase activity, transferring glycosyl groups | 154 |
| 38 | GO:0003743 | Translation initiation factor activity | 151 |
| 39 | GO:0004252 | Serine-Type Endopeptidase Activity | 151 |
| 40 | GO:0051082 | Unfolded protein binding | 148 |

**Supplementary Table 3 -** Top 40 transcripts Gene Ontology statistics of **Biological process** in cluster bean cultivar RGC-1025.

| S No | GO ID | GO Description | Clustered Transcripts |
| --- | --- | --- | --- |
| 1 | GO:0006355 | Regulation of transcription, DNA-templated | 606 |
| 2 | GO:0006412 | Translation | 605 |
| 3 | GO:0005975 | Carbohydrate metabolic process | 397 |
| 4 | GO:0015074 | DNA integration | 297 |
| 5 | GO:0007165 | Signal transduction | 249 |
| 6 | GO:0006886 | Intracellular protein transport | 221 |
| 7 | GO:0006457 | Protein folding | 176 |
| 8 | GO:0045454 | Cell redox homeostasis | 174 |
| 9 | GO:0071555 | Cell wall organization | 174 |
| 10 | GO:0006351 | Transcription, DNA-templated | 167 |
| 11 | GO:0006281 | DNA repair | 154 |
| 12 | GO:0015031 | Protein transport | 146 |
| 13 | GO:0006511 | Ubiquitin-dependent protein catabolic process | 145 |
| 14 | GO:0007018 | Microtubule-based movement | 137 |
| 15 | GO:0016192 | Vesicle-mediated transport | 136 |
| 16 | GO:0055085 | Transmembrane transport | 125 |
| 17 | GO:0006629 | Lipid metabolic process | 115 |
| 18 | GO:0006979 | Response to oxidative stress | 99 |
| 19 | GO:0009734 | Auxin-activated signaling pathway | 95 |
| 20 | GO:0006260 | DNA replication | 92 |
| 21 | GO:0048544 | Recognition of pollen | 91 |
| 22 | GO:0006486 | Protein glycosylation | 90 |
| 23 | GO:0009058 | Biosynthetic process | 90 |
| 24 | GO:0030001 | Metal ion transport | 84 |
| 25 | GO:0006396 | RNA processing | 82 |
| 26 | GO:0006096 | Glycolytic process | 76 |
| 27 | GO:0000398 | mRNA splicing, via spliceosome | 72 |
| 28 | GO:0006952 | Defense response | 71 |
| 29 | GO:0015979 | Photosynthesis | 70 |
| 30 | GO:0006099 | Tricarboxylic acid cycle | 68 |
| 31 | GO:0006310 | DNA recombination | 68 |
| 32 | GO:0006468 | Protein phosphorylation | 66 |
| 33 | GO:0006397 | mRNA processing | 65 |
| 34 | GO:0030244 | Cellulose biosynthetic process | 65 |
| 35 | GO:0016042 | Lipid catabolic process | 64 |
| 36 | GO:0031047 | Gene silencing by RNA | 63 |
| 37 | GO:0015986 | ATP synthesis coupled proton transport | 63 |
| 38 | GO:0009451 | RNA modification | 62 |
| 39 | GO:0006633 | Fatty acid biosynthetic process | 58 |
| 40 | GO:0006357 | Regulation of transcription from RNA polymerase II promoter | 57 |

**Supplementary Table 4 -** Top 40 transcripts Gene Ontology Statistics of **Cellular content** in cluster bean cultivar RGC-1025.

| S. No | GO_ID | GO Description | Clustered Transcripts |
| --- | --- | --- | --- |
| 1 | GO:0016021 | Integral component of membrane | 6584 |
| 2 | GO:0005634 | Nucleus | 2062 |
| 3 | GO:0005737 | Cytoplasm | 706 |
| 4 | GO:0005840 | Ribosome | 518 |
| 5 | GO:0005886 | Plasma membrane | 390 |
| 6 | GO:0000943 | Retrotransposon nucleocapsid | 331 |
| 7 | GO:0005623 | Cell | 299 |
| 8 | GO:0005739 | Mitochondrion | 263 |
| 9 | GO:0009507 | Chloroplast | 243 |
| 10 | GO:0016020 | Membrane | 237 |
| 11 | GO:0005829 | Cytosol | 193 |
| 12 | GO:0005576 | Extracellular region | 152 |
| 13 | GO:0005874 | Microtubule | 146 |
| 14 | GO:0005789 | Endoplasmic reticulum membrane | 140 |
| 15 | GO:0000139 | Golgi membrane | 127 |
| 16 | GO:0005783 | Endoplasmic reticulum | 114 |
| 17 | GO:0005794 | Golgi apparatus | 113 |
| 18 | GO:0005743 | Mitochondrial inner membrane | 92 |
| 19 | GO:0005730 | Nucleolus | 80 |
| 20 | GO:0005618 | Cell wall | 77 |
| 21 | GO:0016459 | Myosin complex | 73 |
| 22 | GO:0048046 | Apoplast | 73 |
| 23 | GO:0009535 | Chloroplast thylakoid membrane | 73 |
| 24 | GO:0016592 | Mediator complex | 65 |
| 25 | GO:0000786 | Nucleosome | 61 |
| 26 | GO:0009570 | Chloroplast stroma | 57 |
| 27 | GO:0015935 | Small ribosomal subunit | 53 |
| 28 | GO:0015934 | Large ribosomal subunit | 50 |
| 29 | GO:0005774 | Vacuolar membrane | 46 |
| 30 | GO:0009506 | Plasmodesma | 44 |
| 31 | GO:0000145 | Exocyst | 43 |
| 32 | GO:0070469 | Respiratory chain | 42 |
| 33 | GO:0005694 | Chromosome | 41 |
| 34 | GO:0005802 | Trans-Golgi network | 40 |
| 35 | GO:0005852 | Eukaryotic translation initiation factor 3 complex | 38 |
| 36 | GO:0043231 | Intracellular membrane-bounded organelle | 38 |
| 37 | GO:0005759 | Mitochondrial matrix | 38 |
| 38 | GO:0005681 | Spliceosomal complex | 37 |
| 39 | GO:0005887 | Integral component of plasma membrane | 35 |
| 40 | GO:0000502 | Proteasome complex | 34 |

**Supplementary Table 5 -** Top 40 KEGG Pathway statistics of cluster bean cultivar RGC-1025.

| S. No | Pathway ID | Name of the Pathway | Transcripts Count |
| --- | --- | --- | --- |
| 1 | 04131 | Membrane trafficking | 1679 |
| 2 | 03036 | Chromosome and associated proteins | 1401 |
| 3 | 04147 | Exosome | 1276 |
| 4 | 02000 | Transporters | 1154 |
| 5 | 04121 | Ubiquitin system | 927 |
| 6 | 03029 | Mitochondrial biogenesis | 896 |
| 7 | 03019 | Messenger RNA biogenesis | 853 |
| 8 | 03011 | Ribosome | 723 |
| 9 | 03400 | DNA repair and recombination proteins | 719 |
| 10 | 03010 | Ribosome | 700 |
| 11 | 01001 | Protein kinases | 688 |
| 12 | 01002 | Peptidases | 645 |
| 13 | 03041 | Spliceosome | 634 |
| 14 | 01009 | Protein phosphatases and associated proteins | 597 |
| 15 | 03009 | Ribosome biogenesis | 573 |
| 16 | 99980 | Enzymes with EC numbers | 546 |
| 17 | 03110 | Chaperones and folding catalysts | 544 |
| 18 | 03000 | Transcription factors | 536 |
| 19 | 01003 | Glycosyltransferases | 503 |
| 20 | 03021 | Transcription machinery | 494 |
| 21 | 04626 | Plant-pathogen interaction | 477 |
| 22 | 04812 | Cytoskeleton proteins | 438 |
| 23 | 04141 | Protein processing in endoplasmic reticulum | 386 |
| 24 | 04075 | Plant hormone signal transduction | 385 |
| 25 | 03016 | Transfer RNA biogenesis | 355 |
| 26 | 04144 | Endocytosis | 350 |
| 27 | 03013 | RNA transport | 347 |
| 28 | 03040 | Spliceosome | 339 |
| 29 | 00190 | Oxidative phosphorylation | 330 |
| 30 | 03012 | Translation factors | 327 |
| 31 | 03032 | DNA replication proteins | 297 |
| 32 | 00010 | Glycolysis / Gluconeogenesis | 294 |
| 33 | 0050 | Starch and sucrose metabolism | 283 |
| 34 | 04016 | MAPK signaling pathway - plant | 269 |
| 35 | 04120 | Ubiquitin mediated proteolysis | 269 |
| 36 | 03015 | mRNA surveillance pathway | 248 |
| 37 | 03051 | Proteasome | 212 |
| 38 | 03018 | RNA degradation | 211 |
| 39 | 00940 | Phenylpropanoid biosynthesis | 204 |
| 40 | 00520 | Amino sugar and nucleotide sugar metabolism | 203 |
